# Cellular Immune Reactions of the Sunn Pest, *Eurygaster integriceps*, to the Entomopathogenic Fungus, *Beauveria bassiana* and Its Secondary Metabolites

**DOI:** 10.1673/031.011.13801

**Published:** 2011-10-19

**Authors:** Arash Zibaee, Ali Reza Bandani, Reza Talaei-Hassanlouei, Davide Malagoli

**Affiliations:** ^1^ Department of Plant Protection, College of Agriculture, University of Guilan, Rasht 41635-1314, Iran; ^2^ Department of Plant Protection, College of Agriculture and Natural Resources, University of Tehran, Karaj 31584, Iran; ^3^ Department of Biology, University of Modena and Reggio Emilia, Via Campi 213/D, 41125, Modena, Italy

**Keywords:** fungi, hemocyte, nodule formation, phagocytosis

## Abstract

In this study, five morphological types of circulating hemocytes were recognized in the hemolymph of the adult sunn pest, *Eurygaster integriceps* Puton (Hemiptera: Scutelleridae), namely prohemocytes, plasmatocytes, granulocytes, adipohemocytes, and oenocytoids. The effects of the secondary metabolites of the entomopathogenic fungus *Beauveria bassiana* on cellular immune defenses of *Eurygaster integriceps* were investigated. The results showed that the fungal secondary metabolites inhibited phagocytic activity of *E. integriceps* hemocytes and hampered nodule formation. A reduction of phenoloxidase activity was also observed. The data suggest that *B. bassiana* produce secondary metabolites that disable several immune mechanisms allowing the fungus to overcome and then kill its host. This characteristic makes *B. bassiana* a promising model for biological control of insect pests such as *E. integriceps.*

## Introduction

Insects live in different environments where they are exposed to various potential invaders such as pathogens, parasites, and parasitoids. However, insects are successful in colonizing every niche on Earth. Thus, their success must also be attributed to their ability to neutralize pathogen invasions (Dunn 1986; Lowenberger 2001; Silva et al. 2002). Insects have a highly efficient immune system that is able to withstand challenges from the majority of microorganisms present in the different habitats where they live. Insect innate immune system is highly developed and it relies on the humoral and cellular components (Gillespie et al. 1997; Lavine and Strand 2002). Humoral responses include the synthesis of a broad spectrum of antimicrobial proteins (Bulet et al. 1999) and the prophenoloxidase (PO) cascade (Ashida and Brey 1998; Cerenius and Söderhall 2004), as well as the production of reactive intermediates of oxygen and nitrogen (Lavine and Strand 2002). Antimicrobial proteins and other immune-related molecules are mainly secreted by the larval hemocytes and fat body (Bulet et al. 1999). Circulating hemocytes have important roles in immune mechanisms of insects against microorganisms (Russo et al. 2001). Insects have several types of hemocytes that are commonly identified by morphological, histochemical, and functional characteristics (Gupta 1985). The most common types of hemocytes reported in the literature are prohemocytes, granulocytes, plasmatocytes, adipohemocytes, and oenocytoids. These five hemocyte types have also been described in many insect species (Gupta 1985). They additionally act through various processes including phagocytosis, nodule formation, and encapsulation to entrap and clear pathogens from the hemolymph. Phagocytosis, the process by which cells engulf large particles from the environment, is essential for host defense against infectious microorganisms and for the clearance of apoptotic cells generated during development (Borges et al. 2008). Nodulation is a complex multi-step process that occurs quickly after microbial infection. Nodule formation is initiated with the micro-aggregation of hemocytes, which entrap large numbers of microorganisms. These micro-aggregates grow in size by recruiting additional hemocytes (Ratcliffe and Rowley 1976). Finally, the process ends with melanization into darkened nodules, which attach to the body wall or to various internal organs (Franssens et al. 2006). In these processes, POs have an important role especially in nodule formation encapsulation. POs are present in the host cuticle and catalyze the hydroxylation of mono and diphenols to quinone intermediates. In insects, the products of the PO cascade are believed to be involved in wound healing, sclerotisation of cuticle, and recognition and melanization of foreign particles (Soderhall and Aspan 1993; Sugumaran 1998). PO-derived quinone and melanin have been shown to have fungistatic and fungicidal activities *in vitro* (Söderhall and Ajaxon 1982; St. Leger et al. 1988; Gillespie et al. 2000).

Also, because of their highly efficient immune system, some insect pests prosper in human-influenced environments such as agro-ecosystems and cause sever economic damage. As a consequence, pests are the target of different control procedures; principally pesticides, and secondarily biological control agents like pathogens and parasitoids. Biological control of insect pests is considered as a priority to decrease side effects due to the use of chemical pesticides. Insect pathogens and entomopathogenic fungi have an ability to overcome the robust immune systems of insects and reach successful pathogenesis (Gillespie et al. 1997; Bandani 2005). Life cycles of entomopathogenic fungi are associated with the synthesis and secretion of several numbers of toxic metabolites including extracellular enzymes, proteins, and low molecular weight compounds such as toxins (Bandani 2005). The growth of the entomopathogenic fungus *Beauveria bassiana* in the hemolymph of the host is associated with the secretion of metabolites, especially those originating from proteins (Mazet et al. 1994; Clarkson and Charnley 1996; Bandani et al. 2000; Bandani 2005). These peptides, such as destruxins and efrapeptins, are indicated as secondary metabolites to differentiate them from the cuticle-degrading protease that favors the invasion of the pathogen. The secondary metabolites are considered to be important pathogenicity determinants (Bandani et al. 2000; Bandani 2005; Zibaee et al. 2009).

Studies on mechanisms of fungal pathogenesis and insect immune responses may provide strategies for the development of more efficient mycoinsecticides for destructive pests. One such insect, the sunn pest, *Eurygaster integriceps* Puton (Hemiptera: Scutelleridae), is a key constraint on wheat production in the wide area of the Near and Middle East, Eastern and Southern Europe and North Africa. *E. integriceps* causes severe damages to the vegetative growth stage of wheat, and significantly decreases both the quantity and quality of grains. Hence, the aims of this study were the identification of distinct morphological types of hemocytes by light microscopy, and the determination of the effects of *B. bassiana* strain B1 and its secondary metabolites on the cellular immune reactions of *E. integriceps.*

## Materials and Methods

### Insects

The insects were collected from the Karaj wheat farm and reared on seeds of the Fallat wheat cultivar in the laboratory at 27 ± 2 °C and 14:10 L:D (Zibaee and Bandani 2010). Insects were fed seeds, and a piece of cotton soaked with water was used as a water source.

### *B. bassiana* culture

*Beauveria bassiana* isolate B1 was cultured at 25 ± 1 °C on Sabouraud Dextrose Agar (pH = 5.6) amended with 1% yeast extract. After 14 days, conidia of *B. bassiana* were washed off with a 0.01% aqueous solution of Tween 20 (Sigma Aldrich, www.sigmaaldrich.com), and different concentrations of spores were prepared as required after several preliminary tests.

### *B. bassiana* toxin extraction

Conidia were harvested from 14-day-old sporulating cultures of *B. bassiana* by scraping the surface with a spatula and suspending the conidia in sterile 0.01% v/v aqueous Tween 20 and diluting to 10^6^ conidia per mL. One mL of conidial suspension was then used to inoculate 100 mL of Czapek Dox (Oxoid, www.oxoid.com) broth supplemented with 0.5% w/v Bactopetone (Oxoid) in 250 mL Erlenmeyer flasks. The fungus was then cultured at 23 °C in a cooled orbital incubator at 1200 g for 12 days. The broth was filtered through four layers of cheesecloth followed by Whatman No. 1 filter paper (Whatman, www.whatman.com) to ensure removal of conidia and hyphal debris. Culture filtrates were extracted as described by Bandani et al. (2000). This entailed extraction with chloroform, filtration of the solvent phase through Whatman No. 1 (phase separator) filter paper to remove any aqueous residue, and removal of the solvent on a rotary evaporator. The residue was dissolved in acetone, filtered through a cotton plug, and concentrated under a stream of nitrogen at 40 °C. The residue was then weighed and stored at 4 °C.

### Determination of hemocyte types by light microscopy

For this purpose, hemolymph from 10 adult *E. integriceps* was collected carefully from severed front legs with a 50 µL sterile glass capillary tube (Sigma Aldrich). The product was immediately diluted in an anticoagulant solution (0.01M ethylenediamine tetraacetic acid, 0.1M glucose, 0.062M NaCl, 0.026M citric acid, pH = 4.6) as described by Azambuja et al. (1991). Several samples were prepared, including 150 µL hemolymph, 15 µL anticoagulant solution, and 80 µL phosphate buffer. 100 µL of each sample were then cytocentrifuged (Shandon Cytospin II, Thermo Scientific, www.thermoscientific.com) onto slides at 200 rpm for 3 min. Nuclei of cells were stained with Hoechst stain 33342, (Invitrogen, www.invitrogen.com) 10 mM for 10 min before being mounted with Mowiol (Merck, www.syngentacropprotection.com). Images of cells were taken under an Eclipse 90i digital microscope (Nikon, www.nikon.com). The microscope was equipped with a super highpressure mercury lamp and connected to a DS cooled camera head DS-5Mc regulated by ACT-2U software (Nikon).

### Injection of insects with spores and secondary metabolite

Adults (0.53 mg, No. 120) were chilled on ice for 15 min, surface sterilized with 70% alcohol, and then injected with 1 µL of five concentrations (10^4^, 10^5^, 10^6^, 10^7^, and 10^8^ spore/mL) of fungal spores by a 10 µL Burkard syringe (Burkard, www.burkard.co.uk). After injection, adults were transferred to a 9 cm diameter Petri dish with wheat grain to follow the course of the assay. Secondary metabolite of *B. bassiana* was dissolved in dimethyl sulfoxide (DMSO) (Sigma Aldrich) so that DMSO concentration in solution was 0.5% or less. Adults were injected with five concentrations of fungal secondary metabolite (2, 6, 15, 30, 50%) to find lethal dose values to evaluate immune responses. The DMSO (0.5%) injected adults were considered as control.

### The effect of fungal spore and its secondary metabolite on hemocyte numbers

To determine if the injection of fungal secondary metabolite or conidia caused any changes in the total and differentiate hemocyte counts (THC and DHC), adults were injected laterally into the thorax with 1 µL of a 10^6^ spores/mL concentration of *B. bassiana* and 3, 7, and 12% concentrations of fungal secondary metabolite, as well as DMSO (0.5%) as a control. Hemolymph was collected 1, 3, 6, 12 and 24 hours after injection from the control group, sporeinjected, and secondary-metabolite injected adults. Samples of hemolymph from 5 adults were bled into 1 mL of ice-cold anticoagulant buffer in 1.5 mL plastic tubes. The tubes were gently inverted 5 to 7 times to facilitate mixing, and both total and different hemocyte numbers were counted using an improved Neubauer hemocytometer. For each treatment, 30 adults were used and the experiment was repeated twice.

### Effects of fungal spores and secondary metabolite on phagocytosis

Thirty adults were injected laterally into the thorax with 1 µL of a 10^6^ spores/mL concentration of *B. bassiana* and 3, 7, and 12% concentrations of fungal secondary metabolite. Hemolymph was collected in 30, 60, and 120 min after injection. Phagocytic activity was determined by counting the cells containing spores in a Neubauer hemocytometer for spore-injected adults, secondary-metabolite injected adults, and DMSO (0.5%)-injected adults as control. Observations were made on an Olympus phase contrast microscope.

### Effects of fungal spores and secondary metabolite on nodulation

Injections were carried out according to the method described above. Nodulation was assessed at 1, 3, 6, 12, and 24-hour intervals. Adults were chilled on ice, hemolymph was gathered in a capillary tube, and then 200 µL samples in three replicates were poured in a hemocytometer and nodules were counted (Franssens et al. 2006).

### Phenoloxidase activity (PO)

In order to test the effect of *B. bassiana* spores and its secondary metabolites on the PO system in adults of *E. integriceps,* a hemocyte lysate supernatant was prepared after injections. Hemolymph from adults was mixed with anticoagulant buffer and centrifuged at 12,000 rpm for 5 min; the supernatant was discarded and the pellet washed gently twice with a phosphate buffer (pH = 6.5, Leonard et al. 1985). Cells were homogenized in 500 mL of phosphate buffer centrifuged at 12,000 rpm for 15 min, and the hemocyte lysate supernatant was used in PO assays. Samples were pre-incubated with buffer at 30 °C for 30 min before the addition of 50 mL of 10 mM aqueous solution of Ldihydroxyphenylalanin. The mixture was incubated for an additional five min at 30 °C and PO activity was measured in a spectrophotometer at 490 nm. One unit of PO activity represents the amount of enzyme required to produce an increase in absorbance of 0.01 min^-1^ (Dularay and Lackie 1985). Activity in treated assays was compared with that of controls. Assays were done in 3 replicates (n = 3) and the whole experiment was repeated twice. For measurement of PO kinetic parameters, different concentrations of L-dihydroxyphenylalanine; 3, 3.5, 4, 5, 6, 7, 8, 9, and 10 mM were mixed with 20 µL of enzyme solution and read at 490 nm. The Michaelis constant (*K_m_*) and the maximal velocity (*V_max_*) were estimated by SigmaPlot v11 (Systat Software, www.sigmaplot.com) and the results of *K_m_* and *V_max_* were the means ± SE of 3 replicates (n = 3) for each concentration.

**Figure 1.**
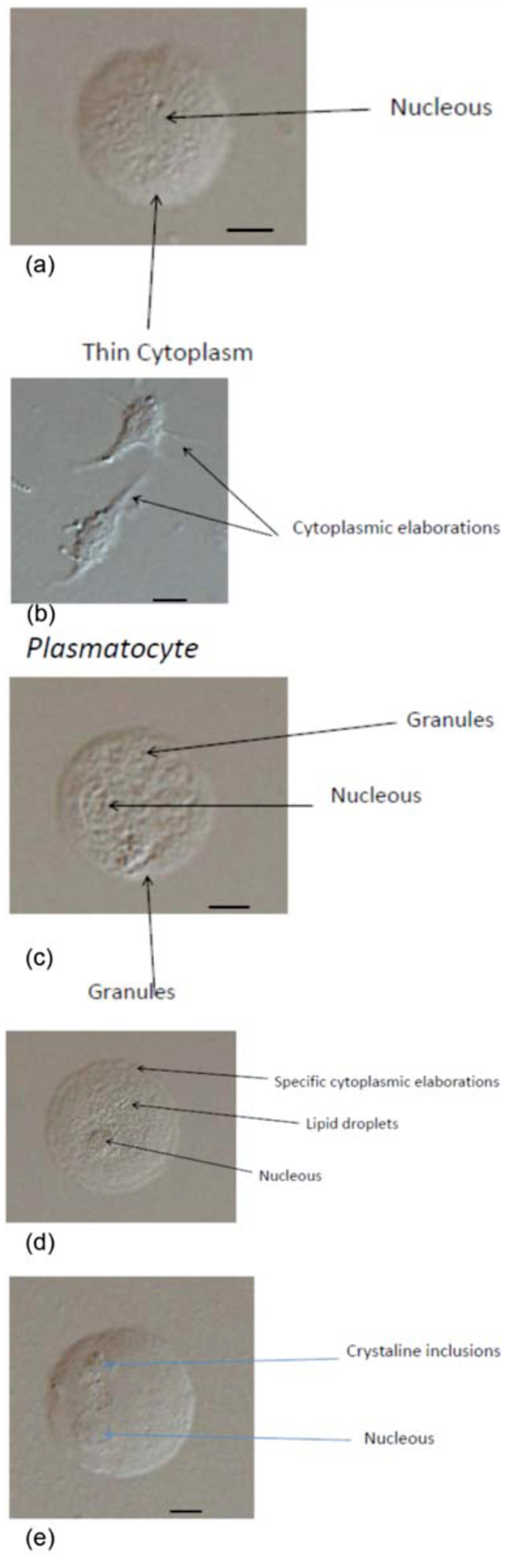
(a–e) Light microscopy of *Eurygaster integriceps* hemocytes: (a) a prohemocyte with a large nucleus (thin arrow) and a thin cytoplasm; (b) a plasmatocyte exhibiting a spindle shape and cytoplasmic elaborations; (c) a granulocyte filled with the typical granules in the cytoplasm (arrow) and large nucleus (arrow); (d) an adipohemocyte with lipid droplets spreading in the cytoplasm and specific cytoplasm elaborations; (e) an oenocytoid with a round eccentric nucleus and crystalin inclusions. Magnification 40× with the exception of (b) (60×). Bar = 5 µm, with the exception of (b) (3 µm). High quality figures are available online.

### Isozyme electrophoresis assay of phenoloxidase

Hemocyte lysate supernatants were prepared 12 h after injections in addition to a control sample using the procedure described by Leonard et al. (1985) and subjected to vertical electrophoresis. Native-Polyacrylamide gel electrophoresis, 4% stacking and 8% separating, was carried out at 100 mV constant current. In addition to provided samples, b-Galactosidase (116 kDa), bovine serum albumin (66.2 kDa), ovalbumin (45 kDa), lactate dehydrogenase (35.5 kDa), restriction endonuclease Bsp 981 (25 kDa), blactoglobulin (18.4 kDa), and lysozyme (14.4 kDa) were used as molecular mass standards.

**Figure 2.**
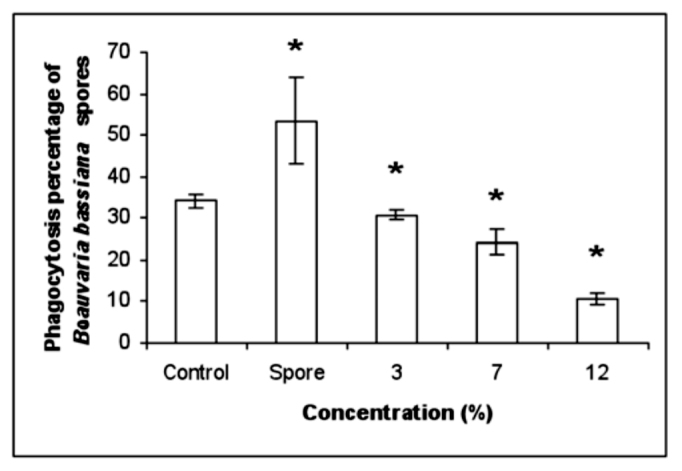
Phagocytosis of *Beauveria bassiana* spores by plasmatocytes. *In vivo* effect of secondary metabolites on the *E. integriceps* phagocytic activity on *B. bassiana* spores. Mean ± standard error, N = 30, *p < 0.05 vs. control. High quality figures are available online.

After electrophoresis, gels were stained by a 10 mM solution of L-dihydroxyphenylalanine, washed with phosphate buffer, and then photographed. Molecular markers were stained by Comassie brilliant blue.

### Protein determination

Protein concentrations were measured according to the method of Bradford (1976), using bovine serum albumin (Bio-Rad, www.bio-rad.com/) as a standard.

### Statistical analysis

POLO-PC software (Leora 1987) was used in the determination of mortality and lethal concentrations. All data were compared by one-way analysis of variance (ANOVA), followed by Tukey's studentized test and line regression analysis when significant differences were found at *p* ≤ 0.05 (SAS 1997). Differences between samplings were considered statistically significant at a probability more than 5% (*p* ≤ 0.05).

## Results

### Identification of hemocytes by light microscopy

Five morphological types of the circulating hemocytes were recognized in the hemolymph of *E. integriceps* adult, including prohemocytes, plasmatocytes, granulocytes, adipohemocytes, and oenocytoids (Figure 1). Prohemocytes are small cells (10–12 µm) with a spherical shape. The nucleus is large, centrally located, and fills the cell so that the cytoplasm occupies just a narrow area around the nucleus (Figure 1a). Plasmatocytes are spindle-like cells with an average size of 22 µm length and 7 µm width, with cytoplasmic processes and granules in the cytoplasm (Figure 1b). Granulocytes (20–25 µm in size) display an oval and regular shape, with a large nucleus and numerous granules in the cytoplasm (Figure 1c). Adipohemocytes are circular cells, 25–30 µm in size, characterized by droplets of lipid spread around the cytoplasm (Figure 1d). Oenocytoids display a round and regular shape (25–27 µm) with a small and eccentric nucleus and very few and small cytoplasmic granules and inclusions (Figure 1e).

**Figure 3.**
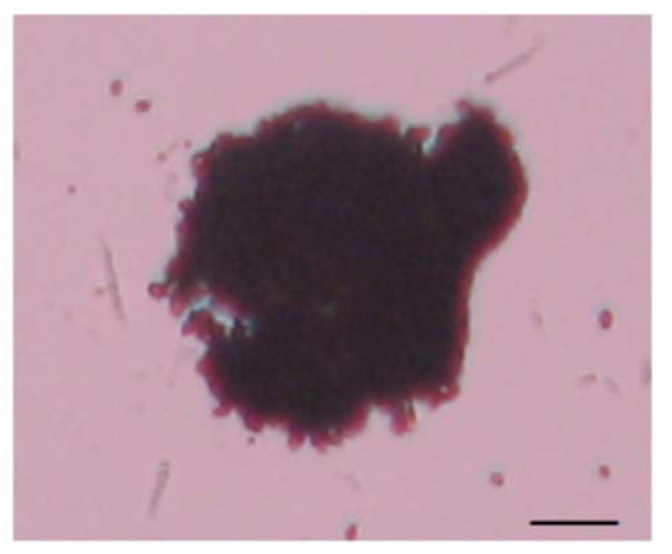
Nodule formation in *Eurygaster integriceps* adults 12 hours after inoculation by *Beauveria bassiana* spores. Scale bar 10 µm. The magnification of images is 40×. High quality figures are available online.

### Effect of *B. bassiana* spores and secondary metabolites on total and count of *E. integricpes* hemocytes

Total and count of hemocytes of adult *E. integriceps* showed significant differences in various intervals after injection of *B. bassiana* spores and secondary metabolites in comparison with control (Tables 1, 2, 3). Table 1 demonstrates that the highest number of hemocytes was observed 6 h after DMSO (0.5%), fungal spores, or secondary metabolites were injected. Increasing of fungal secondary metabolite concentration sharply decreased the total hemocyte number of adults in all intervals after injection and demonstrated a dose-dependent relationship (Table 1). The profile of prohemocytes, plasmatocytes, and granulocytes significantly changed after immune challenge by *B. bassiana* spores after 30, 60, and 120 min intervals when compared with control, while oenocytoids and adipohemocytes showed no significant differences (Table 2). A significant increase in the number of plasmatocytes and granulocytes was observed in injected adults by fungal spores in all intervals after the inoculation, suggesting a possible role in phagocytosis or defense against fungal spores. The number of prohemocytes significantly decreased in response to fungal infection after 60 and 120 min, indicating a potential conversion to plasmatocytes and granulocytes. *B. bassiana* secondary metabolite showed a negative effect on hemocytes of *E. integriceps,* most notably plasmatocytes and granulocytes at different intervals after injection (Table 3). Along with an increase of fungal secondary metabolite concentration, the number of plasmatocytes and granulocytes sharply decreased so that the lowest number was observed for the 15% concentration 30 min after injection (Table 3). However, number of prohemocytes, oenocytoids, and adipohemocytes varied in different concentrations and intervals.

### *In vivo* effect of *B. bassiana* spores and secondary metabolite on phagocytosis

Fungal injection increased phagocytic activity of *E. integriceps* so that it was two times higher relative to the control (DMSO 0.5% injection, Figure 2). Fungal secondary metabolite significantly decreased the amount of phagocytizing cells (Figure 2). The effect of *B. bassiana* metabolite was dose-related, as indicated by the observation that 7 and 12% concentrations had the most relevant effect on the number of phagocytized spores (Figure 2).

### *In vivo* effect of *B. bassiana* spores and secondary metabolite on nodule formation

Fungal spores and secondary metabolite significantly affected nodule formation in adults of *E. integriceps* (Table 4, Figure 3). The highest number of formed nodules was observed three hours after spore injection, and it was significantly higher than that observed in DMSO-injected insects. Injection of fungal secondary metabolite decreased the number of nodules; this decrease was dose-dependent, and the highest effect was observed in 12% concentration for all intervals (Table 3).

**Figure 4.**
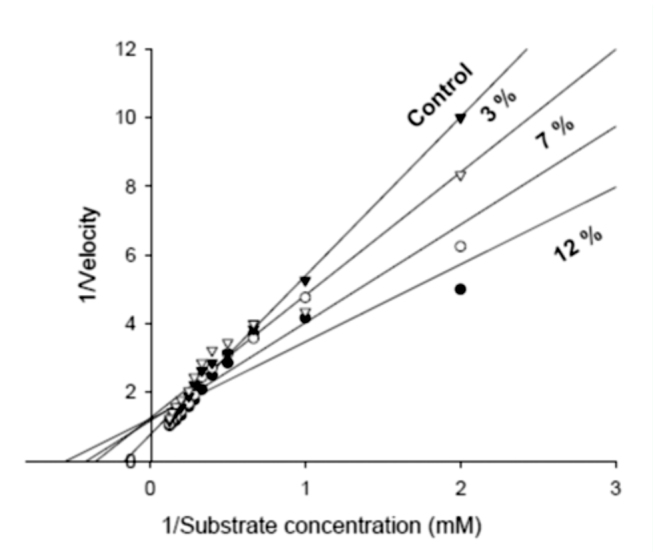
Double reciprocal plot to show the effect of different concentrations of *Beauveria bassiana* secondary metabolites on the phenoloxidase acyivity of *Eurygaster integriceps* adults (I/V_max_ = intercept on the I/V_o_ ordinate, -I/K_m_ = intercept on the negative side of the I/(S) abscissa). EtoH was used as control solution. High quality figures are available online.

### *In vivo* effect of *B. bassiana* spores and secondary metabolite on PO activity

Injecting conidia of *B. bassiana* into *E. integriceps* adults activated the PO system during intervals after inoculation (Table 5). The activity increased during the first 6 hours, at which time the maximum activity was observed. However, although PO activity decreased at 12 and 24 h post-inoculation, it remained significantly different from DMSO-injected controls (Table 5). Fungal secondary metabolite showed a dose-dependent effect, as PO activity decreased along with the increase of fungal secondary metabolite concentrations (Table 5).

**Figure 5.**
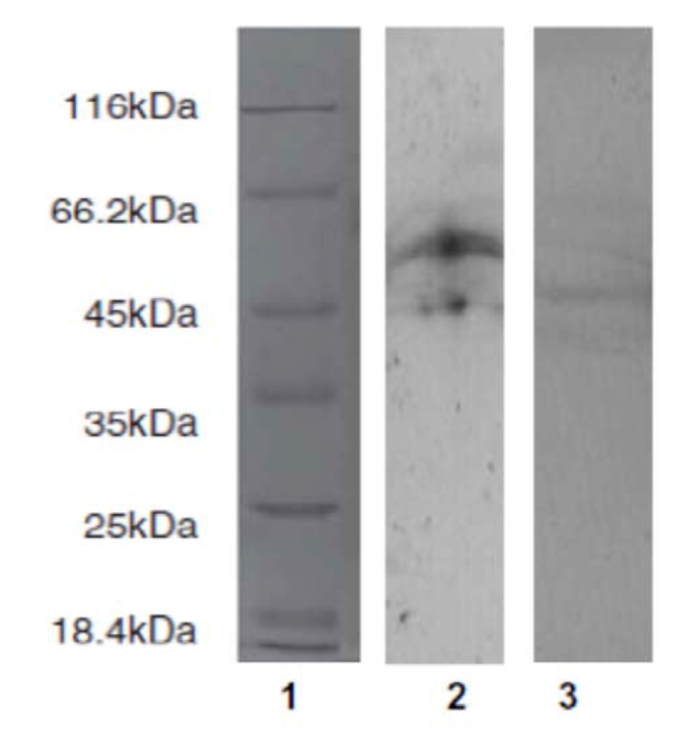
Phenoloxidase isozyme profiles. (1) Molecular marker, (2) control, (3) hemocyte lysate sample after 12-h injection of 15% concentration of secondary metabolite. Samples exposured to *Beauveria bassiana* secondary metabolite for 12 h versus control. High quality figures are available online.

In addition, kinetic parameters of PO activity were also influenced significantly by the different concentrations of *B. bassiana* secondary metabolites (Figure 4, Table 6). PO electrophoresis profiles are shown in Figure 5. Results of gel electrophoresis showed that protein bands in the control sample had estimated molecular masses of 46 and 59 kDa. Twelve hours after the injection, enzyme activity decreased to almost undetectable levels (Figure 5).

## Discussion

This study provides novel information on the hemocyte types of the sunn pest *E. integriceps.* In addition to their identification, total and differentiate counts were performed on hemocyte types. Finally, the effects of *B. bassiana* and its secondary metabolites on the cellular immune reactions of *E. integriceps* were also investigated. *B. bassiana* is the main entomopathogenic fungus in Iran, which has been used against many insect pests across the globe (Wood and Way 1989). Recently, *B. bassiana* was successfully used instead of synthetic pesticides against *E. integriceps,* although the fungal capability to interfere with the immunity of the destructive insect pest was not determined (Talaee and KharraziPakdel 2002).

Results of light microscopy photographing showed that the hemolymph of *E. integriceps* contains five different morphotypes of hemocytes (prohemocytes, plasmatocytes, granulocytes, oenocytoids, adipohemocytes). Similar results were found by Borges et al. (2008) on the blood-sucking bug *Rhodnius prolixus,* where light and transmission electron microscopy demonstrated the presence of five hemocyte types (prohemocytes, plasmatocytes, oenocytoids, adipohemocytes, granulocytes).

*E. integricpes* plasmatocytes and granulocytes are the main actors in phagocytosis of *B. bassiana* spores, and are the most represented among the circulating hemocytes, as observed also in *R. prolixus* (Borges et al. 2008). Injection of spores into the *E. integricpes* hemocyte induced changes in the relative percentage of prohemocytes, plasmatocytes, and granulocytes, suggesting that these cells play an important role in cellular immune response. It has been observed that prohemocytes are located in hematopoietic organs and the hemolymph of several insect species (Borges et al. 2008). Prohemocytes are considered by some authors to be stem cells, from which the other main types differentiate (Yamashita and Iwabuchi 2001; Ling et al. 2005).

In this study, the total hemocyte count (THC) during infection initially increased, then declined. Declines in THC of insects during fungal infection have been recorded previously (Bidochka and Khachatourians 1987; Gunnarsson 1988; Hung and Boucias 1992). The subsequent decline in THC observed in this study may result in part from the formation of nodules induced by soluble fungal metabolites, since there was a significant inverse correlation between THC and nodule counts. However, it is likely that hemocyte aggregation does not fully account for the decline in THC, as cytotoxic fungal metabolites may play an important role. Mazet et al. (1994) found that during infection of moth larvae of *Spodoptera exigua, B. bassiana* produced a toxic metabolite that reduced the activity of hemocytes *in vitro.* Destruxins, produced *in vitro* by the fungus *Metarhizium anisopliae* (Samuels et al. 1988), are toxic to hemocytes (Huxham et al. 1989). Studies on the interaction between *M. anisopliae* and the wax moth *Galleria mellonella* demonstrated that THC in treated larvae was not significantly different from control larvae until 24 h post-injection. However, THC increased in the next few days (Sewify and Hashem 2001). Bandani (2005) observed that THC of *G. mellonella* infected by entomopathogenic fungus *Tolypocladium cylindrosporum* sharply decreased in comparison with control in a dose-dependent fashion. The count of different types of hemocytes indicated that there was an initial increase in plasmatocytes and granulocytes during infection, followed by a decline that included prohemocytes (Bandani 2005). A decline in plasmatocyte number in immunechallenged insects after initial elevation has been noted before (Chain and Anderson 1983; Gunnarsson 1988; Pech and Strand 1996), and may reflect the involvement of plasmatocytes in nodule formation or a particular susceptibility to toxic fungal metabolites as shown in our study. The decline in granulocytes observed may be due to their involvement in the latter stages of nodule formation, as reported for other insects (Gotz and Boman 1985; Pech and Strand 1996; Gillespie et al. 1997).

In insects, different hemocytes may participate in phagocytosis. This study found that plasmatocytes and granulocytes play a critical role in phagocytosis of fungal spores. Injection of fungal secondary metabolites suppressed the hemocyte increase elicited by fungal spores alone. *B. bassiana* secondary metabolites at different concentrations suppress phagocytosis along with nodule formation and PO activity. Thus, it's possible that fungal secondary metabolites interfere with the ligand-receptor interactions, or may cause ultrastructural alteration which interferes with normal hemocyte function (Chen et al. 1998; Vey et al. 2002; Hoffmann, 1994; Hertuet al. 1998).

Insect POs are synthesized as zymogens (prophenoloxidase, proPO), which are activated by proteolytic cleavage at a specific site in response to infection or wounding. Active PO catalyzes the formation of quinones, which undergo further reactions to form melanin (Nappi and Christensen 2005). After a microorganism penetrates into hemocoel, proPO is activated and causes melanization of encapsulated parasites, which is thought to be an important defensive response in insects. PO levels in mycosed insects can be consistently lower than in controls, and declined over the period of infection. In contrast, enhanced hemolymph PO was observed in the grasshopper *Melanoplus sanguinipes* (Gillespie and Khachatourians 1992) and *S. exigua* (Hung and Boucias 1992) injected with spores of *B. bassiana.* Our results are in line with these final observations, since *E. integriceps* proPO activity increased during infection while PO declined. A similar phenomenon has been observed in mosquitoes parasitized by microfilariae (Cho et al. 1998). The activation of proPO system in *E. integriceps* is triggered within minutes after spores penetrate the hemolymph, and the secretion of secondary metabolites by fungus may play an important role to abortion of the activating process. Also, analysis of Lineweaver-Burk plots provides information regarding to the mode of action of *B. bassiana* secondary metabolite against PO activity of *E. integriceps.* The presence of secondary metabolites decreased the value of *V_max_* and increased *K_m_*. The effect of secondary metabolites on the *V_max_* shows that it interferes with the rate of break down of the enzyme-substrate complex. Thus, fungal secondary metabolite inhibits the enzymes by increasing the *K_m_* and decreasing affinity of the enzyme to the substrate. These results show a mixed inhibition by fungal secondary metabolite on PO activity of the sunn pest. In this type of inhibition, fungal secondary metabolites can bind to the enzyme at the same time as the enzyme binds to substrate. This binding affects the binding of the substrate, and vice versa. Although it is possible for mixed-type inhibitors to bind in the active site, this type of inhibition generally results from an allosteric effect, where the inhibitor binds to a different site on an enzyme. Inhibitor binding to this allosteric site changes the conformation (i.e., tertiary structure) of the enzyme so that the affinity of the substrate for the active site is reduced (Stryer 1995; Morris 1978).

## Conclusions

Besides providing the first indications on the *E. integricpes* hemocyte morphotypes, our observations suggest that *B. bassiana* secondary metabolites strongly affect the cellular immune reaction and PO activity of *E. integriceps.* Our experiments indicate that upon an initial stimulation of the insect immune reactions, *B. bassiana* secretes a wide range of secondary metabolites that could have an affect on the host's immune system. Similar results have been demonstrated for toxins of other entomogenous fungi (Vilcinskas et al. 1997; Hung and Boucias 1992; Huxham et al. 1989, Vey et al. 2002). Entomopathogenic fungi are of special importance because they are key regulatory factors in insect pest populations. An understanding of fungal-induced immune responses and the identification of fungal virulence factors and their targets may reveal of significant utility in a biological control scenario.

**Table 1.**
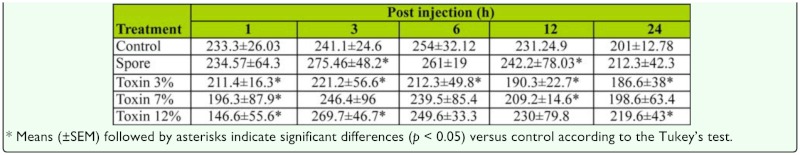
The effect of *Beauveria bassiana* and its secondary metabolites on the total haemocyte count (cells × 10^4^/mL) of *Eurygaster integriceps.*

**Table 2.**
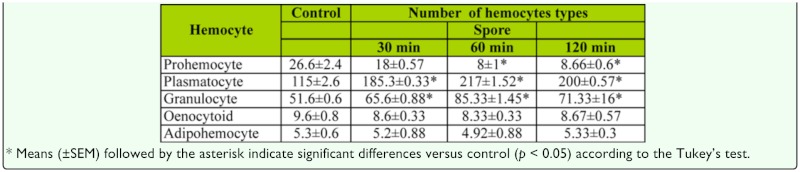
Number of hemocyte types × l0^4^/mL in *Eurygaster integricpes* injected with *Beauveria bassiana* spores.

**Table 3.**
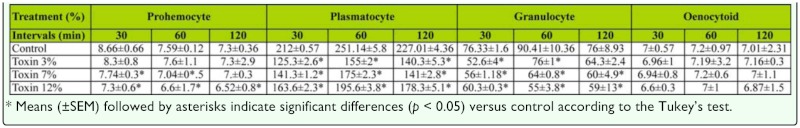
Number of hemocyte types × l0^4^/mL from control and treated insects by secondary metabolites of *Beauveria bassiana* at different times after the immune challenge.

**Table 4.**
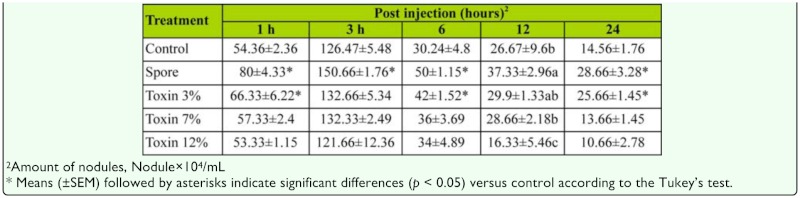
Effects of *Beauveria bassiana* spores and secondary metabolites on the nodule formation of *Eurygaster integriceps* adults.

**Table 5.**
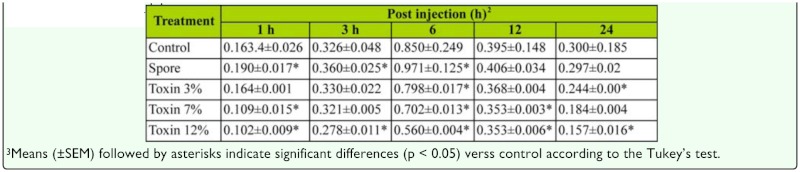
Effects of *Beauveria bassiana* spores and secondary metabolites on the phenoloxidase activity (µmol/min/mg protein) of Eurygaster integriceps adults.

**Table 6.**
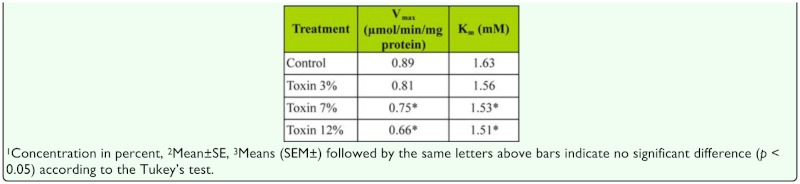
Km (mM) and Vmax (*µmol/min/mg* protein) for phenoloxidase *of Eurygaster integriceps* in the absence and presence of different concentrations of the *Beauveria bassiana* secondary metabolites.

## Abbreviations:

**PO**,: prophenoloxidase;
**THC**,: total hemocyte count
